# The Neural Encoding of Space in Parahippocampal Cortices

**DOI:** 10.3389/fncir.2012.00053

**Published:** 2012-08-17

**Authors:** Lisa M. Giocomo, Yasser Roudi

**Affiliations:** ^1^Kavli Institute for Systems Neuroscience and Centre for the Biology of Memory, Norwegian University of Science and TechnologyTrondheim, Norway

As animals navigate through the environment, they use internal and external sensory cues to update their movements and successfully locate food or nest sites. The rich repertoire of spatial behaviors characteristic of many species has spurred intense research into the neural basis for representing external space. For many years, the mammalian hippocampus has been considered a structure crucial for supporting the creation or storage of spatial information (O’Keefe and Dostrovsky, 1971; Eichenbaum et al., 1999). Yet recent research on other functionally specialized neurons, such as medial entorhinal grid cells, has indicated a wider brain circuit is likely involved in the neurobiology of spatial encoding. Grid cells are neurons that fire in spatially specific locations and form a hexagonal pattern of firing activity that covers the entire environment (Fyhn et al., 2004; Hafting et al., 2005). Despite frequent changes in the animal's running speed and direction, the grid pattern remains periodic, suggesting that the grid population may enable a coordinate system for metric based navigation (Hafting et al., 2005; McNaughton et al., 2006). The discovery of grid cells stimulated a wide variety of experimental and theoretical work aimed at elucidating the neural circuit underlying spatial representations in parahippocampal cortices. In this Research Topic new experimental data, computational modeling, and discussion are presented with a focus on the mechanisms underlying the neural encoding of space. Here, we provide a brief overview of the different types of neurons in the spatial circuit and the relevant context for the papers included in this Topic.

Since the discovery of grid cells, computational modeling has been a key tool in generating hypotheses and theories to explain the origin of periodic and hexagonal firing patterns (For a review see Giocomo et al., 2011; Zilli, 2012). Generally, computational models of grid formation have been grouped into one of two classes; oscillatory interference models and network attractor models. Oscillatory interference models propose that multiple, velocity driven oscillators combine to generate periodic patterns. Attractor network models depend on excitatory or inhibitory recurrent activity and use velocity signals to move a bump of activity across a neural sheet of grid cells. Models in both of these broadly defined categories have significantly evolved since their first inception (O’Keefe and Burgess, 2005; Fuhs and Touretzky, 2006; McNaughton et al., 2006; Burgess et al., 2007) and recent computational models have integrated a combination of elements from oscillatory interference and network attractor classes (see Hasselmo and Brandon, 2012). As computational work on grid cell formation continues to evolve, classification into two broadly defined groups may oversimplify the complexity of various models. As proposed by Zilli (2012), an alternative perspective may be to classify models based on the mechanisms they use to encode, update, and read out position locations.

A prominent feature of grid cells is their spatial scale, which is organized topographically, increasing progressively from dorsal to ventral medial entorhinal cortex (mEC; Hafting et al., 2005). Grid scale is characterized by two spatial measures; the distance between the grid nodes (spacing) and the size of the grid nodes (field size; Hafting et al., 2005). Soon after the discovery of the topographical organization in grid scale, a series of complementary *in vitro* studies described a myriad of biophysical properties which also showed a strong dorsal-ventral organization in mEC (Giocomo et al., 2007; Garden et al., 2008; Giocomo and Hasselmo, 2008; Boehlen et al., 2010 and for a review see Pastoll et al., 2012). The correlative changes in entorhinal cells at the systems and cellular level has resulted in several computational models utilizing single-cell mechanisms to determine the organization of grid scale (Hasselmo et al., 2007; Burgess, 2008; Navratilova et al., 2011). Some versions of oscillatory interference models have focused specifically on the *in vitro* finding that theta band oscillations measured in single mEC neurons decrease in frequency along the dorsal-ventral axis (Giocomo et al., 2007). These oscillatory interference models utilize elements of the intrinsic oscillatory activity, such as the oscillation frequency, to directly determine grid size and spacing. Barry et al. (2012) reviews how a cholinergic decrease in the frequency of theta band oscillations may mediate the expansion of grid scale observed in novel environments (Barry, et al., 2012), an idea supported by *in vitro* slice recordings showing a decrease in the frequency of the theta band resonance after the application of cholinergic agonists (Heys et al., 2010). However, as an alternative or even complementary mechanism, cholinergic activation may induce a change in grid scale by modulating the temporal spiking or integrative properties of layer II mEC neurons (see Pastoll et al., 2012; Yoshida et al., 2012).

Spatial representations are thought to depend not only on grid cells but also on a multitude of other functionally specialized neurons in the parahippocampus. One of these specialized neuronal classes is head direction cells, which respond strongest when an animal faces a particular direction in the environment, irrespective of their behavior or location at the time (Taube et al., 1990; for a review see Clark and Taube, 2012). In mEC, head direction cells exist independently and co-localized with grid cells (Sargolini et al., 2006). This co-localization of head direction signals with grid cells raises the possibility that these two neural populations may interact to some degree. A paper by Kubie and Fenton (2012) proposes that the head direction signal may support the computation, by grid cells, of serial locations in the direction the animal is facing. While the “prospective coding” modeled by Kubie and Fenton highlights a potential influence of head direction signals onto grid cells, experimental data suggests this relationship may be unidirectional. Whitlock and Derdikman (2012) present new data suggesting that the encoding of direction by head direction cells does not depend on the calculation of position by grid cells. The authors demonstrate that, while the fragmentation of the spatial environment results in a break-down of the hexagonal firing patterns of grid cells, head direction cells retain their preferred tuning direction. The stability of head direction with spatial fragmentation may reflect the hierarchical nature of directional signals, which potentially arise from deep brain structures where vestibular cues, rather than spatial cues, dominate the neural response (see Clark and Taube, 2012).

The apparent flexibility in grid scale under conditions of novelty, experience, or fragmentation of the spatial environment may be reflected by hippocampal place cell signals, which have been proposed as downstream targets for the grid cell output (Solstad et al., 2006). Place cells are neurons in the hippocampus that tend to have higher firing rates when the rat is located in a specific portion of the environment (O’Keefe and Dostrovsky, 1971). Navratilova et al. (2012) presents experimental work showing that the directional selectivity of hippocampal place cells develops with experience. Initially, place cell firing on a linear track is determined by allocentric position. With continued experience however, firing changes to respond to sensory information or behavioral cues, raising the possibility that these place cell representations undergo experience-dependent plasticity.

One way to gain a deeper understanding of how these different spatial signals interact in the parahippocampal circuit is to study and compare their developmental time course. Work in very young animals has already demonstrated that specific features of place cells reach adult levels at an earlier age than grid cells (Langston et al., 2010; Wills et al., 2010). However, once grid cells are present, they contain all of the essential features of the adult grid cell population (Wills et al., 2012). The developmental timeline of place and grid cells is also consistent with new data, in this Topic, suggesting that different types of spatial memories follow dissociated timelines. While memory for objects alone develops at a very young age, memory for more complex associations between objects and spatial locations comes online at a later age (Ainge and Langston, 2012).

This Research Topic provides a unique collection of new data and discussion covering a broad scope of questions aimed at understanding the neural representation of external space. We encourage the reader to sample the full range of articles.
